# Confused Connections? Targeting White Matter to Address Treatment Resistant Schizophrenia

**DOI:** 10.3389/fphar.2018.01172

**Published:** 2018-10-18

**Authors:** Candice E. Crocker, Philip G. Tibbo

**Affiliations:** ^1^Department of Psychiatry, Dalhousie University, Halifax, NS, Canada; ^2^Department of Diagnostic Imaging, Nova Scotia Health Authority, Halifax, NS, Canada

**Keywords:** psychosis, white matter, treatment resistance, treatment refractory, schizophrenia, neuropharmacology, neuroimaging

## Abstract

Despite development of comprehensive approaches to treat schizophrenia and other psychotic disorders and improve outcomes, there remains a proportion (approximately one-third) of patients who are treatment resistant and will not have remission of psychotic symptoms despite adequate trials of pharmacotherapy. This level of treatment response is stable across all stages of the spectrum of psychotic disorders, including early phase psychosis and chronic schizophrenia. Our current pharmacotherapies are beneficial in decreasing positive symptomology in most cases, however, with little to no impact on negative or cognitive symptoms. Not all individuals with treatment resistant psychosis unfortunately, even benefit from the potential pharmacological reductions in positive symptoms. The existing pharmacotherapy for psychosis is targeted at neurotransmitter receptors. The current first and second generation antipsychotic medications all act on dopamine type 2 receptors with the second generation drugs also interacting significantly with serotonin type 1 and 2 receptors, and with varying pharmacodynamic profiles overall. This focus on developing dopaminergic/serotonergic antipsychotics, while beneficial, has not reduced the proportion of patients experiencing treatment resistance to date. Another pharmacological approach is imperative to address treatment resistance both for response overall and for negative symptoms in particular. There is research suggesting that changes in white matter integrity occur in schizophrenia and these may be more associated with cognition and even negative symptomology. Here we review the evidence that white matter abnormalities in the brain may be contributing to the symptomology of psychotic disorders. Additionally, we propose that white matter may be a viable pharmacological target for pharmacoresistant schizophrenia and discuss current treatments in development for schizophrenia that target white matter.

The current evidence based approach to treating psychotic disorders is to identify patients early in the disease process and apply a comprehensive pharmacological, psychological and supportive program to change the disease trajectory and improve outcomes (Addington et al., 2013). Despite the clear improvements in patient prognosis with this approach, there is still a cohort within the early phase psychosis population that will not respond to treatment, reflected also in a similar rate of treatment resistance in more established psychosis. Treatment resistance can cause significant personal, family, and societal burden, requiring increased rates of hospitalizations, longer lengths of stay while in hospital, and significant use of other resources (Revicki, 1999; Kennedy et al., 2014). To improve outcomes in this group it is important to have an agreed definition of treatment resistance as this will allow confidence in the research to further the understanding of the biological underpinnings of treatment resistance, informing future treatment strategies.

Treatment resistance criteria as outlined by Kane et al. (1988), with subsequent modifications following, has been well accepted in both clinical and research spheres. It tends to be the definition most used in clinical and drug trial research and includes a minimum requirement of exposure to two different antipsychotics (from different classes) at adequate dose, each for at least 4–6 weeks, without at least a 20% reduction in either positive or negative symptoms as measured by standardized rating scales, and no period of good functioning within the past 5 years (Kane et al., 1988). This definition was initially tested and applied to patients with established and more lengthy illnesses, and modifications over the years has allowed its use in all phases of illness. A recent suggestion, to encompass early phase of illness and to move away from the focus on chronicity, is to define “clozapine eligibility” rather than treatment resistance (see, Williams et al., 2017). The Kane criteria however encompasses the clinical picture of persistent illness and continuous symptoms despite adequate treatment.

Other definitions of lack of treatment responsiveness used in research tend to be variants of the Kane criteria and can include failure to respond to two consecutive rounds of pharmacotherapy of adequate duration (6–8 weeks) and dosage (between 400 and 600 mg per day chlorpromazine equivalents) (Suzuki et al., 2012; Howes et al., 2017). Another commonly used criterion is from the American Psychiatric Association which defines treatment resistance simply as a lack of significant symptom improvement following at least two different trials of antipsychotic medications at therapeutic doses with each treatment round lasting at least 6 weeks (Lehman et al., 2004). A third paradigm that is often used for defining treatment resistance uses the criteria for remission from the Schizophrenia Working Group (Andreasen et al., 2005). In this approach, those individuals who have not met criteria for sustained remission are defined as treatment resistant. Interestingly, a new consensus guideline on terminology has been released from the APA but it has not yet been used in any published research studies (Howes et al., 2017).

While similar, the variations in the criteria used have led to differences in estimations of the prevalence of treatment resistance. Treatment resistant patients may constitute approximately 23% of patients in first episode or early phase populations with the additional observation that 84% of these individuals are potentially treatment resistant from onset of treatment (Demjaha et al., 2017). The percentage of individuals with treatment resistance in later phase or chronic schizophrenia ranges from 5 to 50% but generally a value of roughly 30% is accepted (Juarez-Reyes et al., 1995; Essock et al., 1996; Meltzer, 1997; Lehman et al., 2004; Howes et al., 2017). Within the treatment resistant population, an additional 10–20% may be considered ultra resistant and this is usually defined by a resistance to clozapine treatment (Kane et al., 1988; Juarez-Reyes et al., 1995; Essock et al., 1996).

The current gold standard for treatment of pharmacoresistance is clozapine (Van Sant and Buckley, 2011; Elkis and Buckley, 2016). Despite some uncertainty around the best timing for clozapine initiation, clozapine use in treatment resistant schizophrenia is evidence based and thus reflected in standards of care around identification and treatment of treatment resistance (e.g., National Institute for Health and Care Excellence, 2014; Abidi et al., 2017). Response rates of 60–77% are seen in patients though there are questions regarding its superiority and low rates of use in early phase patients (Williams et al., 2017; Thien et al., 2018). While response rates to clozapine are significant, they are not 100%, indicating that there exists another population of patients with schizophrenia that are resistant even to clozapine. Individuals with schizophrenia may come to a treatment resistant state by being inherently resistant to treatment, while others may lose the effectiveness of antipsychotics after multiple relapses. Importantly, while there are potential gains with clozapine in individuals with treatment resistance, these gains may not be actualized for some individuals who are not be able to tolerate clozapine due to its side effects. The potential for agranulocytosis requires regular blood monitoring and side effects such as sedation, weight gain, and hypersalivation acts as barriers to its use (Mortimer et al., 2010). Clearly more options are needed to address pharmacotherapy in schizophrenia, including more options in treatment resistant cases.

## Rationale for a focus on brain white matter (WM) in treatment resistance

There is not a large body of research examining possible mechanisms behind the development of pharmacoresistance (reviewed in Gillespie et al., 2017). The existing studies have identified several possible mechanisms with a focus on the examination of neurotransmitter systems, which have informed the pharmacology of current therapeutic strategies. The dopaminergic, glutamatergic and serotonergic systems have all been studied in this regard with the dominant dopamine hypothesis underlying the development for many of the current antipsychotics. However, while the dopamine hypothesis, and for that matter other neurotransmitter systems may play a role in treatment resistance (Lau et al., 2013), these systems do not fully explain treatment-resistant schizophrenia (Demjaha et al., 2014; Mouchlianitis et al., 2016a).

Despite uncertainty regarding the underlying causes of schizophrenia and certainly treatment resistant schizophrenia; there are features of the pathophysiology of psychotic disorders that may inform new targets for pharmacological intervention. Schizophrenia has been proposed to be a dysconnectivity syndrome based on cognitive and functional fMRI research (Stephan et al., 2009; Friston et al., 2016). The major connections within the brain are seen structurally as white matter tracts (WM), myelinated axons that move signals between the hemispheres, lobes and gyri of the brain. There is a growing body of evidence, including our own work and the work of others (Palaniyappan et al., 2013; Iwabuchi et al., 2015; Kumar et al., 2015; Crocker et al., 2017), suggesting that a disturbance in neuronal connectivity between different brain regions, rather than abnormalities restricted to individual brain regions, may be responsible for the clinical symptoms and cognitive dysfunctions observed in psychosis (Zhang et al., 2013). This raises the important question of not only the role of WM in the pathophysiology of psychosis, but its role in the more difficult clinical setting of treatment resistance. Inherent in this discussion is the subsequent pharmacological focus on WM as a potential treatment target.

There is not an extensive body of WM neuroimaging studies completed to date in treatment resistant psychotic patients. In this review, we examine the existing literature with respect to WM changes in the context of psychosis and treatment resistance. While this is not a large body of literature and more research clearly needs to be done, existing studies can be used to inform a discussion of how we may be able to use these findings to re-focus our efforts for effective pharmacological treatments to ultimately improve treatment response. Both animal and human studies that have investigated pharmacological WM targets will be discussed.

## Method

This is a narrative review examining the possible role of WM in treatment resistant schizophrenia and its putative utility as a therapeutic target. However, elements of systematic review structure were used to ensure that the literature was comprehensively searched for research around this topic.

### Search strategy

The databases searched were Pubmed, PsycINFO, and Web of Science. For the location of papers examining white matter in treatment resistant patients; search terms included schizophrenia or psychosis and treatment resistance or pharmacoresistant or refractory. Results were then further refined by searching for white matter, connectivity or myelination or diffusion tensor imaging or MRI or magnetic resonance imaging or voxel based morphometry. For articles related to pharmacological treatments and genetics involved in white matter; search terms included white matter and treatment resistant or treatment response or refractory. Articles in both English and French were included in the searches. Ninety-Five Relevant articles and conference proceedings published between 1995 and 2018 were identified. References and abstract listings were screened for eligibility. No abstract proceedings were included in the final literature set. Then all identified studies underwent title and abstract screening followed by full text review. Further articles were identified by scrutinizing the reference lists of included articles. Inclusion criteria were English or French Language, a defined published/peer reviewed criteria for characterization of treatment resistance and specific inclusion of white matter measures.

## An overview of the evidence for WM changes being related to psychosis symptomology

WM abnormalities have been shown to be affected in schizophrenia, including connectivity changes. However, what is the evidence that WM changes correlate with psychosis in individuals responding to treatment and its symptomology? This topic alone could constitute a review article, but to give context for the work in treatment resistant patients, we touch on the key points here. Comprehensive coverage of this topic can be found in recent reviews by others (Dietsche et al., 2017; Parnanzone et al., 2017).

It may first be instructive to consider the mechanisms by which WM can be altered in adult individuals. Myelination begins after 30 weeks gestation but occurs mainly in the post-natal period and is largely complete by young adulthood. Over the past couple of decades extensive research has been done to show that myelination is a dynamic process in the adult brain (Wang and Young, 2014; Almeida and Lyons, 2017), a process that can be affected by various mechanisms. There are thus changes in white matter myelination and oligodendrocytes that occur in adults and have been reported to malfunction in various disease states. Neuroinflammatory processes may lead to WM damage, most often associated with multiple sclerosis resulting in T2-hyperintense lesions that tend to be reduced in number and volume in interferon-beta treated patients (Kaunzner and Gauthier, 2017). The degree of myelin ensheathing has also been associated with abnormal processing speed in studies examining cognitive function in individuals undergoing chemotherapy (Matsos et al., 2017). In examining some of the neurotransmitter systems thought to be involved in schizophrenia, both glutamate and dopamine signaling have been found to have effects on WM. Excitotoxic damage to white matter by glutamate excitotoxicity is another phenomenon that can damage WM and has recently been reported to be associated with vesicular glutamate release (Doyle et al., 2018). Dopaminergic signaling itself is also associated with activity dependent myelination (Roy et al., 2007). Not only does the thickness of the myelin coating on axons affect conductance speed but synaptic activity influences the activity and replacement of oligodendrocytes in the brain throughout life (Almeida and Lyons, 2017). While the overall plan of WM tracts may not be altered after adolescent brain development ceases, there is evidence that fine tuning of the pathways may continue as myelin internodes continue to be created into adulthood (Wang and Young, 2014; Saifetiarova et al., 2017; Snaidero and Simons, 2017).

White matter may play a role in schizophrenia in several ways. First there is the neurodevelopmental hypothesis for schizophrenia which posits that mis-wiring the cortex including the WM connections is the underlying pathology of schizophrenia, to be clear, wholesale rewiring of WM is not likely to be affected by the treatments that are being proposed here (Fatemi and Folsom, 2009). Processes such as those outlined in the previous paragraph may be dysfunctional in schizophrenia though. There is evidence from genetic and post-mortem studies of the interaction with white matter and schizophrenia. DISC1 (disrupted-in-schizophrenia-1) which was identified from genetic studies of a large cohort in Scotland (Zhang et al., 2006), is now known to negatively affect differentiation of oligodendrocyte precursor cells (OPCs) into oligodendrocytes (Hattori et al., 2014). Post-mortem tissue from the dorsolateral prefrontal cortex has been shown in several studies to have downregulated expression of genes that relate to the function of myelin and oligodendrocytes (Hakak et al., 2001; Aston et al., 2004). Neuregulin 1 (NRG-1), a gene involved in regulating oligodendrocyte development and function is also implicated in schizophrenia (Papaleo et al., 2016; Mostaid et al., 2017) There is some evidence for neuroinflammation playing a role in schizophrenia as inflammatory mediators such as IL-10 as a promoter polymorphism in this gene has been shown to be a risk factor for schizophrenia development (He et al., 2006).

Changes in gray matter and brain volume have been well studied in schizophrenia. Less well studied is the trajectory of potential WM changes in the disorder over time (Dietsche et al., 2017). Early studies focused on changes in WM by examining the amount of WM in the brains of affected individuals using structural magnetic resonance imaging (MRI) scans. This early research approach suffered from two main issues. One, most of the studies were done at 1.5T where WM to gray matter junctions can be blurred leading to inaccurate quantitation (Chu et al., 2016, 2017). Second, our current understanding about connections within the brain suggests that dramatic changes in WM are not needed to affect integrity of neural pathways and network nodes within the brain (Rutgers et al., 2008; Englander et al., 2013; De Marco et al., 2017). For these reasons, not observing changes in WM volume on structural MRI does not assure that WM is not affected.

**Figure 1:**
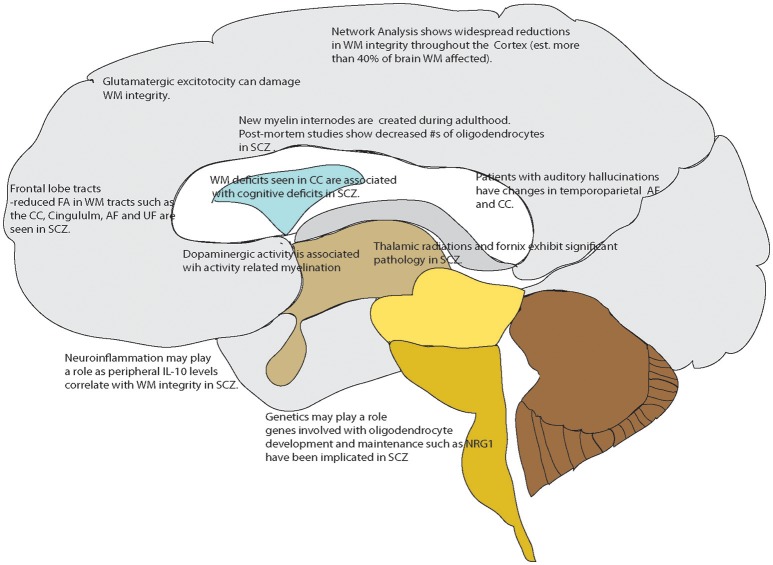
Overview of White matter changes reported in schizophrenia or related to mechanisms known to be affected in schizophrenia that could serve as drug targets. Points referenced in the text.

Current research is focused on using specialized magnetic resonance imaging techniques including diffusion tensor imaging and WM mapping techniques such as T1 mapping and R1 mapping. Diffusion Tensor Imaging (DTI) is often used to examine human *in vivo* WM non-invasively as it can provide an index of the cellular level integrity of WM tissue (Beaulieu, 2002). This neuroimaging method is based on water diffusion, with the variable of fractional anisotropy (FA) indicating a preferred direction of water diffusion in the region of interest (Mori and Zhang, 2006; Nucifora et al., 2007). When FA is found to be reduced in a disease condition, it broadly suggests reduced WM integrity (Ruest et al., 2011). Another technique that addresses the above is T1-weighted/T2-weighted imaging (T1-w/T2-w) using specialized sequences such as MPRAGE and MC-DESPOT (Glasser and Van Essen, 2011); in addition to fast scanning times (important in clinical studies) there are correlations with myelin content with low inter-subject variability (Glasser and Van Essen, 2011; Ganzetti et al., 2014). More recently when examined with a network based analysis, diffusion tensor imaging has shown overall decreases in FA that were widespread; greater than 50% of the cortico-cortical and cortico-subcortical white matter tracts were damaged in patients with schizophrenia and schizoaffective disorder (Klauser et al., 2017). Additionally, there are studies demonstrating an association of deficits in white matter integrity with core cognitive deficits including processing speed in treatment responding patients (Karbasforoushan et al., 2015; Kochunov et al., 2017) Additionally, work in non-treatment resistant patients shows WM alterations in the fornix connections suggesting a mechanism by which WM changes could affect memory (Fitzsimmons et al., 2009; Abdul-Rahman et al., 2011) So, while not clear if white matter changes are a cause or an effect of some underlying pathology, it is clear that white matter integrity is affected in schizophrenia. An overview of these processes that may play a role, or importantly be a subsequent treatment target in schizophrenia, are shown in Figure 1.

We have some evidence that WM changes may be related to changes over time with disease progression in non-resistant patients. The focus on the role of white matter in psychotic disorders is a recent development based on improvements in imaging techniques. There are studies that have examined correlations between WM integrity and the presence of various symptoms present in psychotic disorders (Dietsche et al., 2017; Parnanzone et al., 2017). In patients without treatment resistance, there is evidence that the degree of WM abnormalities is correlated with severity of positive symptoms, primarily reported in DTI and combined structural MRI and DTI studies (Chan et al., 2010; Bracht et al., 2014; Whitford et al., 2014, 2015). Thus, suggesting a direct relationship between white matter integrity and disease course. Examples that are relevant to treatment resistance but did not study it directly, include a recent DTI study that has shown cingulum bundle WM changes in chronic schizophrenia that may be associated specifically with persistent delusions (Oestreich et al., 2016). Another recent study showed severity of psychotic symptoms in hospitalized patients was related to reductions in WM volume measured by T1 structural MRI imaging in the medial portion of the left superior frontal area (Banaj et al., 2018). WM changes were also reported to be present at the onset of illness and potentially able to differentiate the FEP trajectory between what will be schizophrenia or not schizophrenia (Keymer-Gausset et al., 2018). Though treatment resistance was not specifically examined in this study, this could be evidence of how integral WM is to the disease process in schizophrenia. T1 mapping has recently been applied successfully in chronic schizophrenic with results correlating to clinical measures (Iwatani et al., 2015). A recent DTI study in patients at different points in their illness discovered evidence for progressive deterioration of connections over disease course (Di Biase et al., 2017). Additionally, work done with fMRI suggests that the functional brain networks that support higher order cognitive ability in individuals with schizophrenia undergo accelerated aging (Sheffield et al., 2016). As we believe functional networks are underpinned by structural networks, there is a basis for considering that the more damaged the WM, the worse the clinical course. In the next section, we consider the direct evidence for this assumption.

## What evidence do we have for WM changes in schizophrenia being related to treatment resistance?

Neuroimaging research has predominantly examined gray matter in relation to treatment resistance in schizophrenia (reviewed in Mouchlianitis et al., 2016b). Our literature search for papers that examined WM in treatment resistant patients resulted in finding studies that generally measured white matter changes as an adjunct to examination of gray matter content. Overall our search resulted in 15 papers relevant to the topic (Table 1). These papers are neuroimaging studies utilizing the methodologies of structural MRI, diffusion imaging, and T1 mapping. The variety of approaches taken and the small sample sizes in many of these studies highlight the need for further research on this potential new target for treatment of pharmacoresistance in schizophrenia. However, this body of literature overall shows potential for targeting WM in treatment.

**Table 1:** List of White matter and Pharmacoresistance Clinical Studies identified.

**Authors (publication year)**	**Method**	**Comparison done**	**Summary of relevant findings**
Hoptman et al., 2005	Structural MRI (1.5T T1-weighted IR prepared SPGR sequence or T1-weighted MPRAGE). Volumetric analysis	49 resistant to symptoms	Larger orbital frontocortex WM volumes bilaterally were associated with higher aggression scores on the overt aggression scale.
Mitelman et al., 2006	Diffusion Tensor Imaging (1.5T 7 directions, 7.5 mm non isotropic). Fractional Anisotropy (FA). TBSS.	53 resistant 51 responding 41 healthy controls	Right hemisphere showed FA reductions in resistant compared to responders.
Mitelman et al., 2007	Diffusion Tensor Imaging (1.5T 7 directions, 7.5 mm non isotropic). Fractional Anisotropy (FA). Tract analysis by ROI coordinates.	53 resistant 51 responding 41 healthy controls	Differences were seen between responders and resistant in corpus callosum and bilaterally in the fronto-occipital fasciculus. Other changes in left hemisphere only in optic radiation, and rostral segment of anterior limb of internal capsule. Right hemisphere were associated tracts were associated with more PANSS positive scores and negative symptoms inversely associated decreased FA both hemispheres.
Molina et al., 2008	Structural MRI (1.5T T1-weighted 3D gradient echo sequence). ROI volumetrics.	30 resistant 19 responding 44 healthy controls	Increased WM in TR at baseline relative to R and HC in frontal, parietal and occipital lobe. Longitudinal imaging done in a subset (25-28 mo interval between two scans) saw significant decrease in WM in TR relative to R in same lobes as above.
Mitelman et al., 2009	Structural MRI (1.5T T1-weighted 3D SPGR) and diffusion imaging (7 directions)	For structural 65 schizophrenia 16 healthy controls For DTI 17 resistant 17 responder 15 healthy controls	TR patients at baseline had a smaller, and more elongated corpus callosum and lower average FA. During 4 year follow-up, CC in TR patients declined in size but a smaller decline in FA than responders.
Sun et al., 2009	Structural MRI (1.5T T1-weighted IR prepared SPGR sequence). ROI volumetrics.	42 resistant 45 resistant major depressive disorder (MDD) 30 healthy controls	Corpus callosum divided into 5 equidistant segments. Differences in segments were observed between groups and this was interpreted as suggestive of aberrant intrahemispheric connections.
Luck et al., 2011	Diffusion Tensor Imaging (1.5T, 60 directions, 2.2 mm isotropic). FA.	24 resistant 20 responder 30 healthy controls	Resistant had greater decrements in FA in the uncinate fasciculus (UF) and superior longitudinal fasciculus (SLF) as compared to responders and healthy controls. FA values in SLF inversely correlated to several negative symptoms in PANSS. FA correlated to blunted affect only in UF.
Maller et al., 2012	Structural MRI (1.5T T1-weighted IR prepared SPGR sequence). ROI volumetrics.	52 resistant schizophrenia (TR) 182 resistant major depressive disorder (MDD) 76 healthy controls	TR had significantly less whole brain WM as compared to HC and MDD patients (*p* < 0.000).
Holleran et al., 2014	Diffusion Tensor Imaging (1.5T 64 directions, 2.5 mm isotropic). TBSS. Fractional Anisotropy (FA), axial diffusivity (AD) and radial diffusivity (RD) measures.	19 resistant (clozapine naïve) 19 healthy controls	Significantly reduced FA (and increased RD) in the genu, body, and splenium of the corpus callosum, the right posterior limb of the internal capsule, right external capsule, and the right temporal inferior longitudinal fasciculus. Decrease in splenium correlated to illness duration.
Reis Marques et al., 2014	Diffusion tensor imaging (3T 32 directions, 2.4 mm isotropic). Baseline and 12 week followup scans. FA and TBSS.	33 resistant 30 responders 52 healthy controls	Resistant lower FA than both responders and healthy controls. Most pronounced in uncinate, cingulum, and corpus callosum. FA increased in both patient groups with antipsychotic treatment. FA values correlated with PANSS total.
Anderson et al., 2015	Structural MRI (3T T1-weighted MPRAGE). Voxel based morphometry.	19 resistant 15 ultra-resistant 18 responders 20 healthy controls	Whole brain voxel based morphometry showed significant differences in 2 comparisons with responders having significantly less WM than controls (*p* < 0.019) and ultra-treatment resistant having significantly less than HC (*p* < 0.007).
Psomiades et al., 2016	Diffusion tensor imaging (1.5T, 24 directions). FA and tractography examined.	26 resistant with auditory verbal hallucinations 12 resistant with persistent negative symptoms but no hallucinations	FA values were significantly higher in the left arcuate fasciculus (LAF) in resistant patients with hallucinations than in no AVH but negative symptoms resistant patients. Correlation of FA value in the LAF and the severity of auditory verbal hallucinations (*p* < 0.05).
Chen et al., 2018	Diffusion tensor imaging (3T 25 directions). FA and MD	20 resistant 20 responders	Pilot study in First episode patients who were responsive or resistant after 1 year. White matter “impairment” found in right temporal lobe and right occipital lobe. No correlation of decreased FA to symptoms
Huang et al., 2018	3T T1 weighted MPRAGE and diffusion spectrum imaging. Analysis of 76 white matter tracts.	41 resistant 50 responders 50 healthy controls	Differences were found between patient groups and healthy controls for several tracts. Comparison of resistant to responder showed 4 tracts that were significantly different (right fornix, bilateral uncinated fasciculi, temporal pole callosal fibers) further these tracts correlated with negative PANSS scores.
Vanes et al., 2018	T1 mapping (3T mcDespot) Myelin water fraction and cognitive testing	22 Resistant 21 Responsive 24 healthy controls	Resistant and responsive patients showed reduced myelin water fraction compared to HC in bilateral fronto-occipital fasciculi but no difference between patient groups. Callosal Myelin water fraction was associated with cognitive control in patients.

Molina et al. (2008) examined WM structure and volume using MRI in treatment resistant, responsive and health controls (Molina et al., 2008). Thirty patients (21 were male) were treatment resistant using the Kane criteria (Kane et al., 1988). Increased WM content in treatment resistant was seen at baseline relative to responders and healthy controls. Longitudinal imaging done in a subset of their subjects (25–28 month interval between two scans) saw a significant decrease in WM in treatment resistant patients relative to healthy controls, controlling for sex as there was a significant sex difference between groups. However, at follow-up the healthy control group consisted of only 11 subjects (5 women/6 men) and the treatment resistant group 13 subjects (4 females/9 males) which limits their longitudinal findings (Molina et al., 2008). Anderson et al. (2015) examined WM by voxel based morphometry in treatment resistant subjects using the APA 2004 criteria (Lehman et al., 2004) and with an imaging sequence using a higher field strength (3T) magnet that more clearly demarcates the WM to gray matter margins (Anderson et al., 2015). The study reported significant differences compared to healthy controls with responders having significantly less WM than controls (*p* < 0.019) and ultra-treatment resistant having significantly less than HC (*p* < 0.007). While treatment resistant patients trended toward less WM overall, this group had the largest standard deviation and the result did not reach significance (Anderson et al., 2015). Whole brain WM quantitation by structural MRI was also completed comparing 52 treatment resistant schizophrenia patients to 182 treatment resistant major depressive disorder patients and 76 healthy controls. The treatment resistant schizophrenia group had significantly less whole brain WM as compared to HC and MDD patients (*p* < 0.000). This large effect size may have been related to the larger sample size of this study (Maller et al., 2012). The same group published another study examining the structure of the corpus callosum between a subset of the treatment resistant schizophrenia patients, treatment resistant major depressive disorder patients, and healthy controls (Sun et al., 2009). Their analysis divided the corpus callosum into 5 equidistant segments with differences in segments being observed between groups. This was interpreted as being suggestive of aberrant intrahemispheric connections in the treatment resistant group (Sun et al., 2009).

Hoptman et al. (2005) examined WM in the context of overt aggression in a treatment resistant cohort (*n* = 49; 43 M:6 F); controlled for substance use). They reported that larger orbital frontocortex WM volumes bilaterally were associated with higher aggression scores on the overt aggression scale (Hoptman et al., 2005). For this study, treatment resistance was defined as persistence of positive symptoms with typical antipsychotic treatment (600 mg chlorpromazine equivalents or higher) and a functional criteria of a “poor level of functioning” for the previous 2 years was included. This criteria was based on the work of others (Volavka et al., 2002).

T1 WM mapping using a MC-DESPOT sequence has also been reported. Treatment resistant and responsive patients showed reduced myelin water fraction compared to HC in bilateral fronto-occipital fasciculi but there was no difference between the patient groups. Callosal myelin water fraction was also associated with degree of cognitive control during the Stroop task in patients, however there were no difference in the Stroop scores between the two patient groups (Vanes et al., 2018). This study was limited due to small group sizes. There were also no patient demographics given in this paper as the referenced table is missing at time of writing, so it was difficult to judge how ill each patient group was. The groups were divided by a score of at least 4 on at least two of the PANSS (Kay et al., 1987) positive scale for the treatment resistant group and a score of 3 or less on all items of the PANSS for the responder group.

Diffusion tensor imaging studies have reported on treatment resistant patients both in first episode patient populations and more established schizophrenia. Two papers that compared treatment resistant and responder groups with health controls were completed by Mitelman et al. (2006, 2007). One study focused on FA values in 40 Brodmann's areas and the other compared fiber integrity in the same group of patients. Treatment resistance was defined by the criteria of Keefe (Keefe et al., 1987) and tract based spatial statistics (TBSS) were used. TBSS is an automated method of analyzing FA values from different scans by aligning FA values to allow group-wise comparison with a reduction in bias. The right hemisphere of treatment resistant patients showed FA reductions in comparison to patients who responded to treatment, both patient groups had long standing disease and substance abuse (current or historic) was an exclusion criteria (Mitelman et al., 2006). The data from this study was then extended into another paper examining WM tracts. When tracts were compared between treatment resistant and responsive individuals, there were differences in regions of the corpus callosum and bilaterally in the fronto-occipital fasciculus [thought to be involved in semantic processing (Martino et al., 2010)]. The findings in the fronto-occipital fasciculus should be interpreted with caution as the extent and connectivity of this tract is under debate (Bao et al., 2017). Other WM changes were observed in the left hemisphere only and located in the optic radiation, and the rostral segment of anterior limb of internal capsule. Interestingly this group compared changes in WM globally to symptoms as well in their analysis. WM values in the right hemisphere tracts were associated with more PANSS positive scores and negative symptoms were inversely associated with decreased FA in both hemispheres (Mitelman et al., 2007). The same group later performed a longitudinal study again using the criteria of Keefe to define treatment resistance (Keefe et al., 1987) and examined patients with structural and DTI measures (Keefe et al., 1987; Mitelman et al., 2009). In retrospect, we would now be more concerned with the small sample size of this longitudinal DTI study (Melicher et al., 2015) as well as the unbalanced gender ratios; however, the results are quite interesting as they span a 4 year time period comparing treatment responding, treatment resistant and healthy control subjects. The corpus callosum of the treatment resistant patients at baseline was smaller, more elongated and possibly more caudally positioned with lower FA observed in comparison to treatment responsive subjects. Four years later, these non-responders had significant decreases in corpus callosum dimensions but with less decline in FA as compared to responders. This could suggest that dorsoventral thinning was driving the changes in corpus callosum size in treatment resistant subjects and the position changes could be secondary to ventricular enlargement and gray matter loss, as opposed to representing a different arrangement of tracts (Mitelman et al., 2009). Another DTI TBSS study found significantly reduced FA (and increased RD) in the genu, body, and splenium of the corpus callosum, the right posterior limb of the internal capsule, right external capsule, and the right temporal inferior longitudinal fasciculus. Decreased FA in the splenium correlated to illness duration (Holleran et al., 2014) and treatment resistance in this study was defined as failure to respond to two antipsychotic medications (one of which was atypical) and prolonged period of moderate to severe symptoms as defined by the PANSS (Kay et al., 1987). Another short DTI communication found significant differences suggesting WM impairment in the right temporal and occipital lobes in treatment resistant patients as compared to those in remission (Chen et al., 2018). This study defined treatment resistance using China's schizophrenia treatment guidelines and was a pilot study in first episode patients who were responsive or resistant after 1 year of treatment (Chinese Medical Association, 2003). While FA, RD and MD were measured, impairment was not defined other than to say *p* < 0.05 between groups and there was no correlation of decreased FA to symptoms. However, it was not clear how the symptoms were analyzed, for example if the total PANSS score was considered or if subscores were also compared (Chen et al., 2018).

In Psomiades et al. (2016) investigation, two groups of treatment resistant patients were compared, one with auditory verbal hallucinations and one without but with enduring negative symptoms (Psomiades et al., 2016). Treatment resistance was defined in a manner similar to the APA guidelines as the presence of symptoms after two well-conducted antipsychotic drug treatment trials with sufficient doses and duration. FA values were significantly higher in the left arcuate fasciculus (the pathway connecting the frontal lobe with the temporal lobe) in resistant patients with hallucinations as compared to the treatment resistant patients who had enduring negative symptoms (Psomiades et al., 2016). This study brings forward the possibility that WM deficiencies may be specific to the symptomology that is resisting treatment.

There are two other studies that examined WM integrity as it related to treatment outcome in first episode patients. The first study determined resistant status 6 months after scanning and was defined as by the criteria of the remission in Schizophrenia Working group (Andreasen et al., 2005). The treatment refractory group had greater decrements in FA in the uncinate fasciculus (UF) and superior longitudinal fasciculus (SLF) as compared to treatment responders and healthy controls. An exploratory analysis was done to compare diffusion values to FA, and in the SLF an inverse correlation was seen to several negative symptoms from the PANSS (blunted affect, social withdrawal and lack of spontaneity). By contrast, FA correlated to blunted affect only in the UF (Luck et al., 2011). The second study used DTI to try to predict clinical course in first episode psychosis patients. This study used the criteria of remission from the Schizophrenia Working group (Andreasen et al., 2005). The treatment refractory group had lower FA values than both responders and healthy controls at baseline scanning. The decreased values were most significant in the uncinate, cingulum, and corpus callosum. FA values increased in both patient groups with antipsychotic treatment at the follow-up scan. Also FA values negatively correlated with PANSS total scores (Reis Marques et al., 2014). A potential weakness of this study is that the 12 week follow-up for determination of treatment resistance would not reach the threshold for the definition of treatment resistance by other guidelines' criteria (Lehman et al., 2004; Abidi et al., 2017).

A very recent intriguing work was reported by Huang et al. (2018). They used a particular MRI protocol for structural imaging that shows WM very clearly at 3T, which was a T1 weighted MPRAGE in conjunction with diffusion spectrum imaging. Responding and resistant patients were compared to healthy controls as well as each other. Treatment resistance was defined as by the criteria of the remission in Schizophrenia Working group (Andreasen et al., 2005). Analysis of 76 WM tracts was conducted and differences were found between patient groups and healthy controls for several tracts. Comparison of resistant to responder showed 4 tracts that were significantly different (right fornix, bilateral uncinated fasciculi, temporal pole callosal fibers). Further, these tracts correlated with negative PANSS scores.

Complementing the *in vivo* neuroimaging studies already discussed, there is also one paper examining myelination in the substantia nigra in 14 post-mortem samples. Six samples were from treatment resistant subjects and 6 samples were from treatment responsive individuals (a further 2 samples were unknown for treatment response) and these were compared to 9 normal controls. Though this is a small sample size study, tissue from the substantia nigra of treatment resistant patients showed aberrant myelination characterized by increased G ratio (associated with decreased myelin thickness), axons without cytoplasm, and protrusions into the myelin sheath (Walker et al., 2018). The patients in both groups had an average duration of disease of 24 years so while treatment exposure is likely to have been different between groups, these results suggest cellular level changes that may be integral to treatment resistance.

These 15 studies reviewed here are small in number and have methodological differences (including small sample sizes and differences in definition of treatment resistance). However, the signals coming out of this body of work offer the important possibility of WM as another potential target for pharmacological research for schizophrenia as well as treatment resistant schizophrenia.

## Pharmacological WM targets in treatment resistant schizophrenia: animal (preclinical) studies

A challenge of conducting studies in treatment resistant schizophrenia patients is the potential for confounding of results through medication exposure over the disease course. This is an important consideration in neuroimaging of treatment resistant patients who conceivably have had extensive medication exposure and this has not always accounted for in neuroimaging studies to date. An alternative approach to this problem is to examine medication effects directly on white matter in preclinical models. It is worth noting that no animal model fully recapitulates all the symptom domains of schizophrenia but these models allow examination of particular mechanisms of the disease process and potential treatments under controlled conditions.

Exposure of C57BL/6 mice to cuprizone results in demyelination and behaviors that resemble some of those seen in schizophrenia. Cuprizone is a copper chelating agent that when included in the rodent diet for weeks will result in mice developing widespread demyelination, oligodendrocyte loss and myelin breakdown which is similar to changes seen in post-mortem brains of individuals who had schizophrenia (Gudi et al., 2014; Walker et al., 2018). This model was used to test the effects of haloperidol, clozapine, quetiapine on WM recovery and remyelination (Xu et al., 2011). This study examined WM recovery by histological examination using myelin basic protein and anti-glutathione-transferase-pi immunostaining. They found that recovery of WM was still impaired after all three antipsychotic drug treatments with none of the three treatments promoting WM recovery, suggesting that typical and atypical antipsychotics do not act on WM (Xu et al., 2011). This is not surprising given what we know about the mechanism of action of these three drugs and further affirms that an alternative approach to promoting WM recovery is needed. A similar lack of recovery was seen in behavioral testing related to negative symptomology. Potentially this could also help explain why current AP reduce positive symptoms preferentially to negative symptoms. Examining antipsychotic effects directly on WM in preclinical models is important as there is no clear way to balance patients with varying pharmaceutical exposure over the course of their illness in neuroimaging studies, especially in treatment resistant patients who conceivably have had extensive medication exposure.

Reduced numbers of mature oligodendrocytes and increased numbers of microglia are also seen in the cuprizone model of demyelination in C57BL/6 mice (Zhang et al., 2018). This is similar to what is observed post-mortem in people who suffered from schizophrenia (Vikhreva et al., 2016). However, when mice were given N-acetylcysteine, an antioxidant that is a nutrition supplement, at doses of 100 mg/kg/day or greater these reactive immune changes were not seen (Zhang et al., 2018). Changes in interleukin-1 beta and tumor necrosis factor alpha were also significantly decreased with N-acetylcysteine treatment (Zhang et al., 2018). This work is particularly intriguing as a food supplement N-acetylcysteine is readily available and appears to have a wide safety margin. Acetylcysteine is a simple modified amino acid that is used in treating acetaminophen overdose, it has not been associated with serum enzyme elevations during therapy or with episodes of clinically apparent liver injury (National Institutes of Health, 2018). There are some concerns regarding shelf life for the stability of the active ingredients that should be kept in mind, however, as reports show N-acetylcysteine begins to breakdown within 96 h of exposure to air (McEvoy, 2012).

Another preclinical model of schizophrenia is the *St8sia2-/-* mouse (Angata et al., 2004). This mouse model has both behavioral evidence (Krocher et al., 2015) as well evidence from human studies that the gene is part of a susceptibility region with studies that link polymorphisms in the *St8sia2* gene to schizophrenia (Mcauley et al., 2012; Yang et al., 2015; Mandelli et al., 2016). ST8SIA2 is a polysialyltransferase that has as its targets neural cell adhesion molecule 1 (NCAM1) and cell adhesion molecule 1 (CADM1). Polysialylation is a process known to be involved in brain development and ST8SIA2 may be involved in myelin formation as its paralog ST8SIA4 has been shown to do (Koutsoudaki et al., 2010). Examination of myelination and oligodendrocytes in the *St8sia2-/-* mouse model showed lower myelin content, smaller malformed axons and a higher percentage of undifferentiated oligodendroglia (Szewczyk et al., 2017). There may be a role for *St8sia2* in oligodendrocyte differentiation and this could lead to deficits in myelination which in turn can affect axon structure and degeneration. Valproic acid has been shown to downregulate *St8sia2*. This property of valproic acid has been used in experimental autoimmune encephalitis to drive creation of larger numbers of oligodendrocyte precursors (OPCs) and then follow this treatment with Oct4 expressing lentiviral particles which induce differentiation of OPCs, thus resulting in increased numbers of myelinating oligodendrocytes (Dehghan et al., 2016). This is potentially a new approach for promoting myelination in adults and while the focus has been on multiple sclerosis for these types of treatments, the case could be made for treating treatment refractory schizophrenia in the same way. Multiple sclerosis has been shown to be associated with some overlap with schizophrenia symptomology and genetics (Arneth, 2017). Another approach shown in experimental models to improve WM integrity is the injection of extracellular vesicles after experimental stroke in rats (Otero-Ortega et al., 2017). Extracellular vesicles are complexes that are secreted by mesenchymal stem cells after brain insults such as stroke and may hold regenerative properties that are associated with stem cell treatment (Marote et al., 2016). Administration of extracellular vesicles in the rat subcortical infarct stroke model promoted axonal sprouting, oligodendrocyte formation, remyelination, but more importantly tract connectivity was seen (Otero-Ortega et al., 2017). This is potentially a very exciting approach as all of these processes could be useful in repairing the WM changes in treatment resistant schizophrenia.

Another potential avenue for treating WM deficits is suggested by recent work that examined catatonia in both schizophrenia and mice. Catatonia is a psychomotor syndrome that has not been well understood despite being seen across several neuropsychiatric disorders. WM involvement in catatonia is suggested by the association of catatonic signs with reduced expression of 2′-3′-cyclic nucleotide 3′-phosphodiesterase (CNP) which is a myelin associated protein (Hagemeyer et al., 2012). Janova et al. examined the percentage of a loss of function CNP single nucleotide polymorphisms in individuals with catatonia signs within the Gottingen Research Association for Schizophrenia database and found that there was an association between the two (Janova et al., 2018). Extending this work in a preclinical model, catatonia signs could be blocked in *Cnp-/-* mice treated with PLX5622 which pharmacologically blocks colony-stimulating factor 1 receptor and results in glial cell depletion. This group went on to examine WM inflammation in *Cnp-/-* mice by magnetic resonance spectroscopy of a region of the corpus callosum. Myoinositol levels, a marker for glial cell activation, were reduced with PLX5622 treatment in the *Cnp-/-* mice suggesting that neuroinflammation plays a role in the WM processes associated with catatonia (Janova et al., 2018; Pease-Raissi and Chan, 2018). Overall these preclinical studies provide some insight into the possible mechanisms by which WM may play a mechanistic role in schizophrenia and in particular facets of the disorder that are more frequently treatment refractory.

## Pharmacological WM targets in treatment resistant schizophrenia: human studies

**Figure 2:**
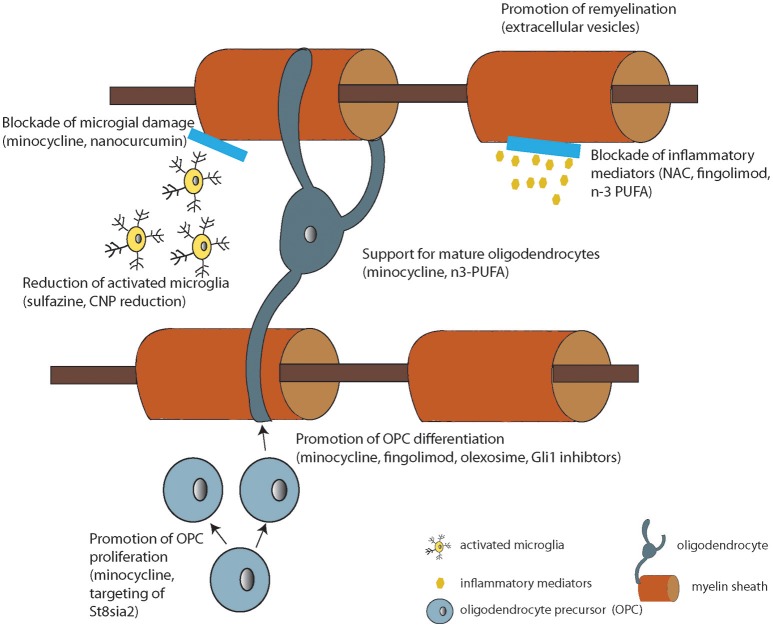
Diagram summarizing the cellular or molecular mode of action of the compounds under development listed in Table 2 (where known).

Based on the literature reviewed here, there are WM deficits that correlate with treatment resistance in schizophrenia. While other mechanisms of pharmacoresistance are still possible for any particular patient, if we consider WM as a target for therapy, there are options that are in development for human use. In fact, myelin enhancing strategies have been under investigation in human subjects for many years as effective treatments for multiple sclerosis are sought. Thus, repurposing and investigating these approved therapeutics currently in use for other medical conditions for treatment resistant patients is a reasonable approach. More specifically, putative myelinenhancing therapies would be potential candidates for large-scale clinical trials in schizophrenia. These include myelin-enhancing agents such as n-3 PUFA (Chen et al., 2014), minocycline (Rodgers et al., 2013), clemastine (Liu et al., 2016), polyphenols (Ghaiad et al., 2017), and potential neuro/myeloreparative agents such as sulfasalazine (Kim et al., 2015), nano-curcumin (Mohajeri et al., 2015), stem cell enhancing therapies such as Gli-1 inhibitors (Samanta et al., 2015), immunodmodulators such as fingolimod [FTY720, approved for use in MS (Kipp and Amor, 2012)], olexosime (Magalon et al., 2016) and retinoid receptor activators such as pioglitazone (Natrajan et al., 2015; Palaniyappan, personal comm.) (Summarized in Figure 2 and Table 2).

**Table 2:** List of potential therapeutics targeting white matter for treatment resistant schizophrenia.

**Compound/drug (IUPAC name)**	**Proposed mode of action**	**Current stage of development relative to psychosis treatment**
Minocycline (2*E*,4*S*,4a*R*,5aS,12a*R*)-2-(Amino-hydroxy-methylidene)-4,7-bis(dimethylamino)-10,11,12a-trihydroxy-4a,5,5a,6- tetrahydro-4*H*-tetracene-1,3,12-trione)	Protection of white matter by blocking microglial damage to white matter, promoting proliferation and maturation of oligdendroglial precursor cells and preservation of mature oligodendrocytes.	FDA approved and available by prescription. Multiple trials with some focus on treatment resistance: “Adjunctive Minocycline in Clozapine Treated Schizophrenia Patients” (clinicaltrials.gov id NCT01433055), “Minocycline Augmentation of Clozapine for Treatment Resistant Schizophrenia” (clinicaltrials.gov id NCT02533232), “The Benefit of Minocycline on Negative Symptoms in Schizophrenia: Extent and Mechanisms” (clinicaltrials.gov id NCT02928965), “Minocycline add-on to Antipsychotics for the Treatment of Negative and Cognitive Symptoms in Schizophrenia” (clinicaltrials.gov id NCT02907437)
Fingolimod (2-amino-2-[2-(4-octylphenyl)ethyl]propane-1,3-diol)	Mimic of sphingosine 1-phosphate (S1P)	FDA approved as a treatment for multiple sclerosis and available by prescription. Phase 2 clinical trial: “Fingolimod in schizophrenia patients (STEP)” (clinicaltrials.gov id NCT0177970)
Pioglitazone (5-[[4-[2-(5-ethylpyridin-2-yl)ethoxy]phenyl]methyl]-1,3-thiazolidine-2,4-dione)	peroxisome proliferator activated receptor gamma (PPARgamma) agonist	FDA-approved as diabetes treatment and available by prescription. Phase 4 trial: “Pioglitazone as a Treatment for Lipid and Glucose Abnormalities in Patients With Schizophrenia” (clinicaltrials.gov id NCT00231894)
N-acetylcysteine (also called NACS; (2R)-2-acetamido-3-sulfanylpropanoic acid)	Antioxidant, shown to reduce interleukin-1 beta and tumor necrosis factor alpha in preclinical demyelination models.	Dietary supplement with multiple trials for psychosis underway: Phase 4 trial for “Treatment of Cognitive and Negative Symptoms in Schizophrenia With N-acetylcysteine” (clinicaltrials.gov id NCT02505477) and Phase 2 “N-Acetyl-Cysteine (NAC) in Early Phase Schizophrenia Spectrum Psychosis” (clinicaltrials.gov id NCT01354132)
Nano-curcumin [(1E,6E)-1,7-bis(4-hydroxy-3-methoxyphenyl)hepta-1,6-diene-3,5-dione]	Reduction in inflammation by several mechanisms including inhibiting NF-kappaB and induction of iNOS, reduces myelin and blood brain barrier damage, may improve microRNA profile	Dietary supplement with clinical trials relevant to treatment resistant schizophrenia: Phase 2 trial “Curcumin as a Novel Treatment to Improve Cognitive Dysfunction in Schizophrenia” (clinicaltrials.gov id NCT02104752), and “Curcumin Addition to Antipsychotic Treatment in Chronic Schizophrenia Patients” (clinicaltrials.gov id NCT02298985).
(n-3) polyunsaturated fatty acids (n-3 PUFA)	Increasing oligodendrocyte membrane integrity and blocking inflammatory damage to myelin	Dietary supplement. One trial found to examine cardiovascular disease which is also relevant to individuals with psychosis “Fish Oil-derived N-3 Polyunsaturated Fatty Acids and Extracellular Vesicles (HI-FIVE)” (clinicaltrials.gov id NCT03203512).
Clemastine fumarate ((2*R*)-2-{2-[(1*R*)-1-(4-chlorophenyl)-1-phenylethoxy]ethyl}-1-methylpyrrolidine)	Enhanced oligodendrocyte progenitor differentiation, may reverse some negative epigenetic changes	FDA approved as an allergy treatment. Available over the counter in most jurisdictions. No clinical trials for psychosis registered currently.
Olexosime {(NZ)-N-[(8S,9S,10R,13R,14S,17R)-10,13-dimethyl-17-[(2R)-6-methylheptan-2-yl]-1,2,6,7,8,9,11,12,14,15,16,17-dodecahydrocyclopenta[a]phenanthren-3-ylidene]hydroxylamine}	Targeting proteins of the outer mitochondrial membrane and prevention of permeability transition pore opening mediated by oxidative stress. Promotion of oligodendrocyte maturation	Was in development for Amyotrophic Lateral Sclerosis (ALS) and Spinal Muscular Atrophy (SMA). Roche announced it was discontinuing development in June 2018. No clinical trials for psychosis registered currently.
Sulfasalazine ((3Z)-6-oxo-3-[[4-(pyridin-2-ylsulfamoyl)phenyl]hydrazinylidene]cyclohexa-1,4-diene-1-carboxylic acid)	Inhibition of CD44v-xCT (cystine transporter, reduction of the number of macrophages and microglia	FDA approved as Antirheumatic and gastrointestinal treatment. Available by prescription. No clinical trials for psychosis registered currently.
Gli1 inhibitors (such as 5-fluorouracil, methotrexate, cisplatin, vismodegib)	Promotes differentiation of stem cells into mature oligodendrocytes	Preclinical development
Extracellular Vesicles derived from stem cells	Promotion of axonal sprouting, oligodendrocyte formation, remyelination with tract connectivity	Preclinical development
Reduced expression of 2′-3′-cyclic nucleotide 3′-phosphodiesterase (CNP)	Blockade of microglial activation	Preclinical development
Targeting of *St8sia2*	Promotion of stem cell production of oligodendrocyte precursors.	Preclinical development

(n-3) polyunsaturated fatty acids (n-3 PUFA) are dietary components and are a family of fatty acids mainly found in oily fish and fish oil supplements. Myelin sheaths are formed from the cell membranes of oligodendrocytes which contain polyunsaturated fatty acids. A sufficient quantity of n-3 PUFA in the diet was found to increase WM integrity and executive function in a study of healthy elderly (Virtanen et al., 2008). There is reason to believe that these fatty acids may also play a role in psychotic disorders. Lower total PUFA concentration in the membranes of erythrocytes was associated with lower fractional anisotropy (FA) measured by DTI in the corpus callosum and bilateral parietal, occipital, temporal and frontal WM in a study in early psychosis patients (Peters et al., 2013). An additional mechanism for how these compounds could help in treatment resistant patients is suggested by work in an Experimental autoimmune encephalomyelitis model of demyelination, n-3 PUFA was able to block the release of inflammatory mediators by microglia and keep the M2 phenotype thus blocking microglial damage to myelin (Chen et al., 2014). Thus n-3 PUFA could help by increasing oligodendrocyte membrane integrity and blocking inflammatory damage to myelin.

Minocycline is in the family of tetracycline antibiotics and is used as an anti-acne treatment. It crosses the blood-brain-barrier and is immunomodulatory which led to it being studied for use in relapsing remitting MS (Rodgers et al., 2013). This immunomodulatory role and the research linking the possibility of negative symptoms with neuroinflammation has led to a number of clinical trials examining minocycline as an add-on to second-generation antipsychotic treatment. A recent meta-analysis of eight RCTs with minocycline used as an adjunctive medication concluded that minocycline appeared to be superior to placebo for positive, negative and general symptom scores, exhibiting a good safety profile (Xiang et al., 2017). Interestingly, minocycline also appears to protect WM by both blocking microglial damage to WM and promoting proliferation and maturation of oligdendroglial precursor cells (Schmitz et al., 2014). It is thus also positioned to be investigated for use in treatment resistant schizophrenia.

Clemastine fumarate is an ethanolamine-derivative, and a first generation histamine H1 antagonist used for allergic rhinitis. Based on its ability to stimulate oligodendrocyte differentiation, clemastine is being considered as a treatment for MS and is in phase 2 testing (Green et al., 2017). One small note is often made in reference to use of clemastine in that similar to other antihistamines, it is known to cause drowsiness. Clemastine is particularly interesting as a possible treatment for treatment refractory schizophrenia as not only does it enhance oligodendrocyte progenitor differentiation but it also causes epigenetic changes which may be important in the overall disease course (Liu et al., 2016).

Polyphenols such as green tea polyphenol mixture (GTPP) and its active ingredient, epigallocatechin-3-gallate (EGCG), prevent both the neurite outgrowth-inhibiting activity and growth cone-collapsing activity of the C-terminal domain of Nogo-A, which is derived from myelin (Gundimeda et al., 2015). Another polyphenol, resveratrol, which is a stilbenoid polyphenol, and known to pass the blood brain barrier, has been shown to reverse cuprizone-induced demyelination, and improved mitochondrial function in preclinical work (Ghaiad et al., 2017). However, results in humans with a 6 month trial of resveratrol examining inflammatory mediators and brain structure have not been impressive to date (Huhn et al., 2018).

Sulfasalazine is a pro-drug that is converted to 5-Aminosalicylic Acid (5-ASA) and is used to treat ulcerative colitis and rheumatoid arthritis. Sulfasalazine treatment promoted remyelination in the CNS of a transgenic zebrafish model of NTR/MTZ-induced demyelination by reducing the number of macrophages/microglia, suggesting an immunomodulatory function of sulfasalazine in remyelination (Kim et al., 2015). Sulfasalazine inhibits the CD44v-xCT (cystine transporter) which is crucial for growth and viability and required for synthesis of intracellular glutathione. CD44+ microglia are involved in neuroinflammatory processes (Matsumoto et al., 2012). However, caution may be in order as trials with this drug in MS patients have shown limited effects and a significant side effect profile (Shirani et al., 2016).

Nano-curcumin is a more bioavailable form of curcumin. Curcumin is a polyphenolic phytochemical that has antioxidant properties. Many claims have been made to its effectiveness for a variety of conditions. However, it has been shown to reduce inflammation by several mechanisms including inhibiting NF-kappaB and induction of iNOS (Xie et al., 2011). In a rat experimental autoimmune encephalomyelitis (EAE) model, polymerized nano-curcumin was shown to reduce myelin damage and blood brain barrier breakdown (Mohajeri et al., 2015). Another particularly intriguing therapeutic target is the ability of nano-curcumin to restore microRNA expression in a small cohort of relapsing-remitting MS patients (Dolati et al., 2018). MicroRNAs have been shown to be involved in both the pathogenesis of schizophrenia, affect drug metabolizing enzymes and are altered by antipsychotic treatment (Swathy and Banerjee, 2017). Curcumin has been tested as an adjunct to fluoxetine in major depressive disorder with no significant improvement in depression scores but also no safety concerns (Sanmukhani et al., 2014). Curcumin may act on several systems to improve treatment response and nano-curcumin makes use of this phytochemical therapeutically possible.

Neural stem cell based approaches are another possible avenue to repair WM. Gli-1 inhibitors act upon a pool of neural stem cells that express Gli1 and when Gli1 expression is repressed, these cells differentiate into oligodendrocytes (Samanta et al., 2015).

Fingolimod [FTY720, an immunodmodulator approved for use in MS (Kipp and Amor, 2012)], is believed to mimic sphingosine 1-phosphate (S1P) *in vivo* and a lipid mediator that acts through G protein coupled receptors and can cross the blood-brain-barrier (Chun and Hartung, 2010). Receptors for S1P are also present in the CNS. There is a listing for phase two clinical trial examining fingolimod in schizophrenia patients (STEP) active in the clinical trial registry that was due to finish recruiting patients in Dec 2017 (See Table 2). A recent study from a phase 3 trial in MS patients showed significantly less ventricular volume enlargement and less WM loss with fingolimod as compared to placebo (Gaetano et al., 2018). This raises a possibility for fingolimod to modify not only WM loss but the pathological ventricular enlargement seen in schizophrenia which would be an exciting possibility.

Olexosime (TRO19622) is a novel cholesterol-oxime mitochondrial-targeted neuroprotective compound that acts by targeting proteins of the outer mitochondrial membrane and prevents permeability transition pore opening mediated by, among other things, oxidative stress. It was originally designed for movement disorders such as Amyotrophic Lateral Sclerosis (ALS) and Spinal Muscular Atrophy (SMA) where it was thought to have potential to prevent mitochondrial rupture (Lenglet et al., 2014). Olesoxime has been shown to promote myelination through action on oligodendrocytes to accelerate maturation and remyelination in preclinical models of demyelination (Magalon et al., 2016).

Pioglitazone, an FDA-approved peroxisome proliferator activated receptor gamma (PPARgamma) agonist, was originally designed as a diabetes drug but has found further targets in MS. As a diabetes drug, it works by increasing the body's sensitivity to insulin, a potential bonus for patients with metabolic issues in addition to psychosis symptomology. Pioglitazone has been shown through diffusion tensor imaging to reduce lesion formation in normal appearing WM in relapsing remitting MS patients (Shukla et al., 2010). It has also been shown to decrease WM lesions in the corpus callosum in a stroke model in hypertensive rats and reduced microglia proliferation in the same model (Lan et al., 2015). Both of which would be beneficial in treatment of WM deficits in treatment resistant patients.

A number of these agents are suitable for drug repurposing and repositioning applications, which greatly enhances the lab-to-clinic transition (Ashburn and Thor, 2004). Repurposing RCTs are already underway for some of these agents [e.g., fingolimod (fingolimod in Schizophrenia clinicaltrials.gov)] and pioglitazone (Iranpour et al., 2016). Of these minocycline, which predominantly limits neuronal damage by promoting oligodendrocyte progenitor proliferation and preserving mature oligodendrocytes (Guimaraes et al., 2010; Schmitz et al., 2012; Ma et al., 2015; Scheuer et al., 2015), and pioglitazone which promotes antioxidant defense of oligodendrocytes (Bernardo et al., 2009) have already shown promise in treating psychosis (Chaudhry et al., 2012; Iranpour et al., 2016). Further work is needed to see if an association exists between extensive WM changes and pharmacoresistance, but if it does then these individuals can be specifically targeted for clinical trials of myeloprotection (Palaniyappan, personal comm.).

## Conclusion

Schizophrenia is highly heterogeneous in presentation leading some to propose, based on symptoms and even genome wide association studies, that we might be better served to re-consider “The Schizophrenias” (Peralta and Cuesta, 2011; Schizophrenia Working Group Of The Psychiatric Genomics Consortium, 2014). The rich tapestry of variation in schizophrenia is a challenge for researchers but is also an opportunity for developing personalized medicine strategies. Differential treatment response is one of these areas of disease variation that may point the way to designing a personalized plan and shed light on the etiology behind the disorder.

Ultimately, a combination of clinical profile, imaging and possibly as we move forward pharmacogenomics will likely be required to identify what therapeutic approach best suits each individual pharmacoresistant patient. The observation that greater than 80% of the individuals who are treatment resistant are already resistant at the first episode psychosis stage of schizophrenia, gives further credence to the idea that there is more than one type of schizophrenia and clearly targeting the dopaminergic system is not sufficient for 30% or greater of the patient population. WM is a viable therapeutic target for this portion of the patient population. The studies cited here are observational so it is unclear if the WM damage is related to a cause of schizophrenia or the result of damage from some underlying metabolic process; however, in either case improvement of WM integrity in pharmacoresistant patients was overall associated with an improvement in symptomology in the studies that examined this outcome (Table 1). Further examination of either repurposing WM modulating drugs from other demyelinating diseases or moving those in preclinical work forward to examine in treatment resistant patients should be a goal moving forward.

## Author contributions

CC and PT wrote the abstract. CC performed the literature search, wrote the first draft and drew the figures. PT edited the manuscript and tables for submission and accuracy. Both authors read and approved the final version.

### Conflict of interest statement

The authors declare that the research was conducted in the absence of any commercial or financial relationships that could be construed as a potential conflict of interest.
