# Corrigendum: Confused Connections? Targeting White Matter to Address Treatment Resistant Schizophrenia

**DOI:** 10.3389/fphar.2018.01417

**Published:** 2018-12-04

**Authors:** Candice E. Crocker, Philip G. Tibbo

**Affiliations:** ^1^Department of Psychiatry, Dalhousie University, Halifax, NS, Canada; ^2^Department of Diagnostic Imaging, Nova Scotia Health Authority, Halifax, NS, Canada

**Keywords:** psychosis, white matter, treatment resistance, treatment refractory, schizophrenia, neuropharmacology, neuroimaging

In the original article, we neglected to include funding section with the funder Canadian Institutes of Health Research (CIHR), grant number 391348, awarded in February 2018, to PT and collaborators on the grant, Dr. Lena Palaniyappan, CC, Dr. Ali Khan, Dr. Jacob Cookey, and Dr. Sherry Stewart. The authors apologize for this error and state that this does not change the scientific conclusions of the article in any way.

In the original article Palaniyappan, personal comm. was not cited in the article. The citation has now been inserted in *Pharmacological WM Targets in Treatment Resistant Schizophrenia: Human Studies*, paragraphs 1 and 11 and should read:

Paragraph one:

Based on the literature reviewed here, there are WM deficits that correlate with treatment resistance in schizophrenia. While other mechanisms of pharmacoresistance are still possible for any particular patient, if we consider WM as a target for therapy, there are options that are in development for human use. In fact, myelin enhancing strategies have been under investigation in human subjects for many years as effective treatments for multiple sclerosis are sought. Thus, repurposing and investigating these approved therapeutics currently in use for other medical conditions for treatment resistant patients is a reasonable approach. More specifically, putative myelinenhancing therapies would be potential candidates for large-scale clinical trials in schizophrenia. These include myelin-enhancing agents such as n-3 PUFA (Chen et al., 2014), minocycline (Rodgers et al., 2013), clemastine (Liu et al., 2016), polyphenols (Ghaiad et al., 2017), and potential neuro/myeloreparative agents such as sulfasalazine (Kim et al., 2015), nano-curcumin (Mohajeri et al., 2015), stem cell enhancing therapies such as Gli-1 inhibitors (Samanta et al., 2015), immunodmodulators such as fingolimod [FTY720, approved for use in MS (Kipp and Amor, 2012)], olexosime (Magalon et al., 2016) and retinoid receptor activators such as pioglitazone (Natrajan et al., 2015; Palaniyappan, personal comm.) (Summarized in Figure 2 and Table 2).

Paragraph 11:

A number of these agents are suitable for drug repurposing and repositioning applications, which greatly enhances the lab-to-clinic transition (Ashburn and Thor, 2004). Repurposing RCTs are already underway for some of these agents [e.g., fingolimod (fingolimod in Schizophrenia clinicaltrials.gov)] and pioglitazone (Iranpour et al., 2016). Of these minocycline, which predominantly limits neuronal damage by promoting oligodendrocyte progenitor proliferation and preserving mature oligodendrocytes (Guimaraes et al., 2010; Schmitz et al., 2012; Ma et al., 2015; Scheuer et al., 2015), and pioglitazone which promotes antioxidant defense of oligodendrocytes (Bernardo et al., 2009) have already shown promise in treating psychosis (Chaudhry et al., 2012; Iranpour et al., 2016). Further work is needed to see if an association exists between extensive WM changes and pharmacoresistance, but if it does then these individuals can be specifically targeted for clinical trials of myeloprotection (Palaniyappan, personal comm.).

The authors apologize for these errors and state that this does not change the scientific conclusions of the article in any way. The original article has been updated.

## Conflict of Interest Statement

The authors declare that the research was conducted in the absence of any commercial or financial relationships that could be construed as a potential conflict of interest.
